# Dengue Fever: Therapeutic Potential of *Carica papaya* L. Leaves

**DOI:** 10.3389/fphar.2021.610912

**Published:** 2021-04-26

**Authors:** Md. Moklesur Rahman Sarker, Farzana Khan, Isa Naina Mohamed

**Affiliations:** ^1^ Department of Pharmacy, State University of Bangladesh, Dhanmondi, Dhaka, Bangladesh; ^2^ Pharmacology and Toxicology Research Division, Health Med Science Research Limited, Dhaka, Bangladesh; ^3^ Department of Pharmacology, Faculty of Medicine, Universiti Kebangsaan Malaysia (The National University of Malaysia), Cheras, Malaysia

**Keywords:** *Carica papaya*, dengue, viral infection, *Aedes aegypti* virus, thrombocytopenia, immunity, dengue treatment, tropical disease

## Abstract

Dengue, a very widespread mosquito-borne infectious disease caused by *Aedes aegypti* virus, has been occurring during the monsoons every year. The prevalence and incidence of dengue fever and death due to its complications have been increased drastically in these recent years in Bangladesh, Philippines, Thailand, Brazil, and India. Recently, dengue had spread in an epidemic form in Bangladesh, Thailand, and Philippines. Although the infection affected a large number of people around the world, there is no established specific and effective treatment by synthetic medicines. In this subcontinent, Malaysia could effectively control its incidences and death of patients using alternative medication treatment mainly prepared from *Carica papaya* L*.* leaves along with proper care and hospitalization. *Papaya* leaves, their juice or extract, as well as their different forms of preparation have long been used traditionally for treating dengue fever and its complications to save patients’ lives. Although it is recommended by traditional healers, and the general public use Papaya leaves juice or their other preparations in dengue fever, this treatment option is strictly denied by the physicians offering treatment in hospitals in Bangladesh as they do not believe in the effectiveness of papaya leaves, thus suggesting to patients that they should not use them. In Bangladesh, 1,01,354 dengue patients have been hospitalized, with 179 deaths in the year 2019 according to information from the Institute of Epidemiology, Disease Control, and Research as well as the Directorate General of Health Services of Bangladesh. Most of the patients died because of the falling down of platelets to dangerous levels and hemorrhage or serious bleeding. Therefore, this paper aims to critically review the scientific basis and effectiveness of *Carica papaya* L. leaves in treating dengue fever based on preclinical and clinical reports. Thrombocytopenia is one of the major conditions that is typical in cases of dengue infection. Besides, the infection and impairment of immunity are concerned with dengue patients. This review summarizes all the scientific reports on *Carica papaya* L. for its ability on three aspects of dengue: antiviral activities, prevention of thrombocytopenia and improvement of immunity during dengue fever.

## Introduction

Dengue infection is transmitted through *Aedes egypti*, a flaviviridae virus (Moreno-Sanchez et al., 2006). Dengue occurs within 5–7 days after an infected mosquito bites a healthy person. The infection symptoms usually include high fever, rash, and headaches as well as muscle and joint pain, eye pain, vomiting, and nausea (Morens and Brody, 2008; Ahmad et al., 2011). Dengue virus has four serotypes (Dengue virus-1 to Dengue virus-4) and a certain individual can be infected multiple times due to the different types of virus. Infection by dengue virus more than once can result in a severe form of dengue known as dengue hemorrhagic fever (DHF) (Morens and Brody, 2008). Dengue is widespread around the world and it has been estimated that there are about 50–100 million cases of dengue fever (DF) and 500,000 cases of dengue haemorrhagic fever (DHF) every year (Ahmad et al., 2011). It was observed by Electron micrographs that the dengue virus has relatively smooth virions with a diameter of approximately 500 Å. It has a plus-sense RNA genome and there are three structural proteins that are core, membrane, and envelope (Rey et al., 1995). Thrombocytopenia is a clinical condition associated with dengue (Lale et al., 2006). The specific mechanism behind thrombocytopenia associated with dengue has not been found yet. It has been speculated that thrombocytopenia in dengue is developed by the downregulation of platelet production in bone marrow, the destruction of existing platelets, the production of antibodies against platelets, and the clearance of platelets mediated by these antibodies (Boo et al., 2019). However, there are currently no available treatments or vaccine against dengue virus and although different explanations are available, the specific way in which a dengue virus can cause dengue fever is yet to be revealed (Nuri and Ming, 2016). From the ancient times, natural plants significantly contributed to the discovery and development of many emerging medicines in almost all of the therapeutic categories (Atanasov et al., 2021). In recent days, phytomedicines, nutraceuticals and herbal medicines have attracted the attention of researchers for extensive investigations with an aim of the discovery and development of medicaments for the treatment of diabetes (Chen et al., 2018; Chen et al., 2019; Munira et al., 2020; Sarker and Soma, 2020), cancer (Sheikh et al., 2017a; Sheikh et al., 2017b; Shajib et al., 2018), autoimmune and immunocompromised conditions (Goto et al., 2010; Sarker et al., 2011; Sarker et al., 2012a; Sarker et al., 2012b; Sarker, 2012; Sarker et al., 2012; Sarker and Gohda, 2013; Sarker et al., 2014; Sarker et al., 2016; Sarker, 2021), inflammation and infectious diseases (Imam et al., 2013; Sarker, 2015; Rouhi et al., 2017; Nesa et al., 2018), hyperlipidemia (Kazemipoor et al., 2015; Sarker, 2015), and neurological and adaptogenic problems (Das et al., 2017; Ibrahim et al., 2020) due to the limitations of conventional drugs because of the development of drug resistance and adverse effects and chronic toxicities in long term treatment. *Carica papaya* L. is an important medicinal plant which has recently been explored for investigations of its medicinal properties and identification of its bioactive compounds and establishment of their mechanism of actions. However, papaya leaves have been traditionally used for the treatment of dengue fever and proven effective in improving thrombocytopenia in *in vitro* and *in vivo* models although the possible mechanism behind this potential is not specified (Dengue and severe dengue, 2019).

Previously several studies were done to review the existing literature on therapeutic potential of *Carica papaya* leaves against dengue associated thrombocytopenia and anti-DENV potential. Bok et al. (2020) in their literature review titled as “The plausible mechanisms of action of Carica papaya on Dengue infection: A comprehensive review” discussed the findings of the existing data from clinical studies. Pentewar et al. (2017) briefly described dengue management, thrombocytopenia manifestation in dengue and role of papaya leaves extract in dengue infection in their literature review titled as “Papaya Leaf Extract To Treat Dengue: A Review”. Two systemic and meta-analyses were made, reviewing the findings of the clinical studies and dosage safety (Charan et al., 2016; Rajapakse et al., 2019). However, no literature review is available that provides a wide picture of dengue infection manifestation and role of *Carica papaya* leaves extract in minimizing the thrombocytopenia.

In this study, we tried to depict the dengue situation in the current world, the mechanism of thrombocytopenia in dengue, the role of papaya leaves extracts and their other preparations in alleviating thrombocytopenia, cytotoxic (lervicidal) activities, and immunomodulating properties with comprehensive study reports from preclinical (*in vitro* and *in vivo*) and clinical study reports. Besides, other plants with anti-dengue effects with the same constituents and toxicity study reports of Papaya leaves extracts or other preparations were also extensively reviewed in this paper.

**FIGURE 1:**
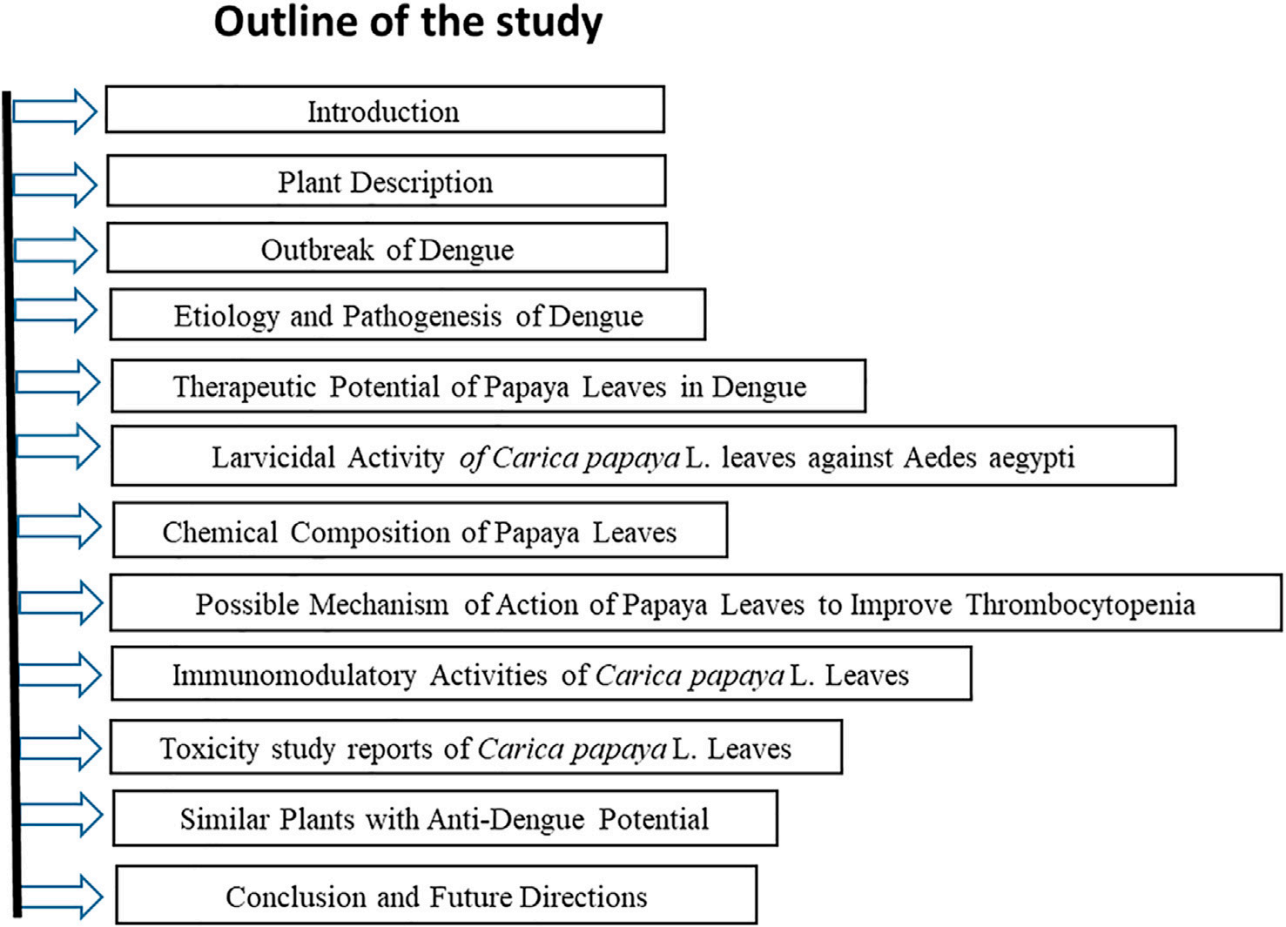
Outline of the Study: Therapeutic Potential of *Carica papaya* L. Leaves against dengue fever.

Above all, the mechanism of thrombocytopenia in dengue and the role of papaya leaves in alleviating this condition as well as studies available to investigate the therapeutic role of papaya leaves in dengue are reviewed extensively. A outline of the current review on the therapeutic potential of *Carica papaya* L. Leaves against dengue fever has been illustrated in Figure 1.

## Plant Description

*Carica papaya* L. is commonly known as papaya, papaw, or pawpaw in Australia, Mamao in Brazil, Pepe in Bangladesh, Papeeta in Hindi, and several other names in different countries of the world. The papaya tree is fast-growing, semi-woody, produces latex, and is usually short-lived. The cultivars are distinguished on the basis of the number of leaf main veins, types of stomata, color of the leaf petiole, number of lobes at the leaf margins, leaf shape, and structure of the wax on the leaf surface (da Silva et al., 2007; Sekeli et al., 2018). *Carica papaya* L. is mostly cultivated in tropical and subtropical lowland regions such as Hawaii, Australia, and South-East Asia. India, Mexico, Indonesia, Thailand, and Nigeria are the largest papaya growing countries among the developing countries (Reddy et al., 2009; Lopes et al., 2019). Historically, papaya was found to be distributed from Mexico to the Panama, and spread to the Caribbean and Philippines during the exploration of the Spanish explorer, Don Francisco Marine, in the 16th century, and later on spread to Malaysia, India, and other Asean countries from the Philippines (Nakasone and Paull, 1998; Fitch, 2005; Sekeli et al., 2018). Carica papaya L. belongs to the Caricaceae family. It has a lifespan of about five–ten years and grows as a singular unbranched trunk. It is an herbaceous perennial plant, has a milky latex and it can grow up to 12 m in height. Its fruits weigh between 1,000 and 3,000 g and produces fruits all year round. The leaves are up to 75 cm wide, palmately lobed, have hollow petioles and the blades are divided into five to nine segments. The flowers are born on inflorescences and are present in the axils of the leaves. *Carica papaya *L*.* fruits are melon-like, have smooth, green skin that looks yellow or orange when ripe, and usually have more than 1,000 seeds (Krishna et al., 2008; Abdulazeez and Sani, 2011).

The taxonomy of the plant (*Carica papaya* L.) is listed below (Habtemariam, 2019):

**Table:** 

Family	Caricaceae
Genus	*Carica* L
Species	*Carica papaya* L

Papaya is consumed worldwide either as a vegetable or fresh fruit or in processed products like jam, preserved and canned (Sekeli et al., 2018). Traditionally, different parts of the plant are used in the treatment of various diseases. The white latex of papaya contains an enzyme called “papain” and it is used to tenderize meat, as an antiseptic for wound dressing, abortifacient, in case of dyspepsia, ringworm, psoriasis, and cancerous growth (Pandey et al., 2018). The root infusion has been used traditionally in the treatment of venereal diseases, piles, and yaws (Adeneye, 2014).

## Outbreak of Dengue: Scenario

Dengue incidents have been found to be increasing dramatically in recent years. This disease is now considered endemic in 100 countries by the World Health Organization (WHO) where, before 1970, only nine countries had a severe experience with dengue. Countries with severe cases of dengue are located in the regions of Africa, the Americas, the Eastern Mediterranean, South-East Asia, and the Western Pacific, whereas the most seriously affected countries are in the American, South-East Asian and Western Pacific regions. In 2018, about one to two million cases of dengue incidents were reported in the Americas, South-East Asia and Western Pacific, whereas in 2016, the numbers recorded were 3.34 million (Epidemiological Update Dengue, 2019, Aug 09).

### The Americas

In 2015, the number of cases of dengue incidents in the Americas was 2.35 million, with 1,181 deaths due to dengue (Dengue and severe dengue, 2019). In 2016, the number of dengue incidents reported was 2.38 million with 1,032 deaths. Between the period of 2017–2018, a 73% decrease in dengue incidents was observed. However, an increase in cases of dengue fever has been observed in the year 2019 (Dengue and severe dengue, 2019). According to the Pan American Health Organization (PAHO), in 2019, 2,029,342 cases of dengue with a 0.04% fatality rate (207.9 cases per 100,000 population) were observed in the Americas during the epidemiological week (EW) one to EW 30. Brazil, Colombia, Honduras, and Nicaragua were the four countries with the highest incidents of dengue that year (Epidemiological Update Dengue, 2019, Aug 09). In the first five months of 2020, there were 1.5 million dengue cases detected; among them, Brazil has the highest number of cases.

In the year 2018, a total of 264,262 probable cases of dengue in Brazil were reported with the incidence rate of 126.7 per 100,000 population where in 2017, this incidence rate was at 115.3 cases per 100,000 population (Epidemiological Update Dengue, 2019, Feb 22). However, during the period of EW one and EW 28 in 2019, 1,345,994 probable cases were reported in Brazil where 830,376 cases were confirmed with the incidence of 645.6 cases per 100,000 population. There were 485 cases of fatality due to dengue (Epidemiological Update Dengue, 2019, Aug 09). During EW1 to EW21 in Brazil, 1,040,481 cases were found with an incidence rate of 370.4 cases per 100,000 population (Epidemiological Update Dengue, 2020, June 12).

In 2018, 44,825 cases of dengue were reported in Columbia with 176 probable deaths due to dengue and an incidence rate of 179.9 cases per 100,000 population at-risk (Epidemiological Update Dengue, 2019, Feb 22). Up to the EW30 in 2019, according to PAHO, 71,736 probable cases of dengue were observed in the country with a fatality rate of 0.05% and incidence rate of 267.1 cases per 100,000 population (Epidemiological Update Dengue, 2019, Aug 09). Until June 2020, 54,192 cases were found (Epidemiological Update Dengue, 2020, June 12).

In Honduras, 42,346 cases of dengue were observed as of EW 30 in 2019 with a fatality rate of 0.19% and an incidence rate of 462.39 per 100,000 population. Within the same period of the year, a total of 12,081 suspected cases with 47 deaths due to dengue were reported in Guatemala (Epidemiological Update Dengue, 2019, Aug 09). In 2020 from January until June 17, 940 cases were found (Epidemiological Update Dengue, 2020, June 12).

### Western Pacific Regions

In the western pacific region, dengue is widespread in Cambodia, Malaysia, Lao PDR, the Philippines, and Vietnam. According to WHO, a total of 3,130 dengue incidents have been suspected in Cambodia during the year 2017 (Dengue Update Number 532, 2017). A total of 9,885 dengue cases were observed as of week 51 in the year 2018 in Malaysia (Dengue Update Number 559, 2019). Till week 29 of 2020, 4,450 dengue cases with five deaths were found (Dengue Update Number 601, 2020). In Lao PDR, 10,943 cases of dengue with 14 deaths were reported in 2017 (Dengue Update Number 532, 2017). In the following year of 2018, the total reported cases of dengue incidents were 6,204 until week 51 (Dengue Update Number 559, 2019). Cumulative dengue cases as of week 33 of 2019 were 24,758 including 51 deaths (Dengue Update Number 576, 2019). Until week 32 in 2020, 402 cases of dengue incidents were recorded (Dengue Update Number 601, 2020).

In the year of 2017, a total of 80,805 dengue cases with 169 deaths were reported in Malaysia, which was less than that in 2016 where 97,041 cases with 229 deaths due to dengue were reported (Dengue Update Number 532, 2017). In 2018, dengue cases reported in Malaysia were 80,615 with 147 deaths (Dengue Update Number 559, 2019). As of August 17, 2019, the number of dengue incidents in this country was 85,270 including 121 deaths (Dengue Update Number 576, 2019). By the end of 2019, people reported with dengue fever were estimated to be 119,198 with a death count of 162 (The Star, 2019). As of week 32 of 2020, 1,807 dengue cases were found in Malaysia (Dengue Update Number 601, 2020). In the Philippines, a total of 117,654 dengue cases including 657 deaths were reported until November, which was 38.6% lower than that of the same period in 2016 (Dengue Update Number 532, 2017). In 2018, the number of cases due to dengue in the country were 199,271 (Dengue Update Number 559, 2019). As of August 23, 2019, cases of dengue reported in the country were 208,917 with 882 deaths (Dengue Update Number 576, 2019). During week 29 of 2020, 446 dengue cases with two deaths were reported (Dengue Update Number 601, 2020). In Vietnam, an increased number of dengue cases was observed in 2017 compared to that of the previous year. In 2017, a total of 175,795 cases of dengue incidents with 30 deaths were reported by the WHO (Dengue Update Number 532, 2017). In the year of 2018, 113,850 cases with 16 deaths were reported as of week 47, whereas in week 30 of 2019, 124,751 cases with 15 deaths were reported due to dengue (Dengue Update Number 559, 2019; Dengue Update Number 576, 2019). On week 31, 2020, 2,236 cases were reported from 46 out of 63 provinces with no death record (Dengue Update Number 601, 2020).

### South-East Asia

In this region, Bangladesh, India, Indonesia, Sri Lanka, and Thailand have the highest numbers of dengue incidents. The first epidemic of dengue fever in Bangladesh was recorded in 2000 when 5,551 dengue infections were reported from Dhaka, Chittagong, and Khulna cities with 93 deaths (Rahman et al., 2002). Increased incidence of dengue fever has been observed from 2018 where 10,148 people were infected with dengue and 26 of them died (Alam, 2019). This number of infected people in 2018 was three times more than that of dengue infected people in 2017, which was 2,769 with eight deaths (Cousins, 2019; Rahman, 2019). In 2016, the number of deaths was 14 out of 6,060 dengue patients, and in 2015, six people died out of 3,162 due to dengue. However, no death was recorded among 375 dengue patients reported in 2014 (Rahman, 2019). In the year 2019, Bangladesh struggled with the worst outbreak of dengue fever. According to the Directorate General of Health Services, the disease broke out to epidemic proportions and set a new record of 1,01,354 hospitalizations. The Institute of Epidemiology, Disease Control, and Research reviewed 276 dengue death reports prepared by registered doctors and confirmed that dengue had took 179 lives (Official dengue death toll makes record, 2020). As of July 2020, 331 dengue cases were reported in Bangladesh (Geographical distribution of dengue cases reported worldwide, 2020).

In Indonesia, a total of 59,047 cases of dengue fever were reported with 444 deaths due to dengue in 2017. The incidence rate this year was 0.75% (Harapan et al., 2019). In 2016, the number increased to 204,171 with 1,598 fatalities. Only in January of 2019, 9,634 dengue cases with 100 fatalities were reported (Cahya et al., 2019). About 68,700 cases and 446 deaths, as of June 22, 2020 were reported in Indonesia (Geographical distribution of dengue cases reported worldwide, 2020). In India, according to the National Vector Borne Disease Control Program (NVBDCP), a total of 188,401 cases of dengue were reported in the year 2017 with 325 cases of fatalities. In 2018, the number was reduced to 101,192 cases of dengue fever with 172 deaths (Rai, 2019). However, different states in India have reported an alarming number of dengue cases in 2019. In South India, 6,210 cases of dengue with 6 deaths have been reported on June 21. More than 5,000 cases of dengue with 200 death were observed in Gujarat within June (Kaur, 2019). In Uttarakhand, 1,340 cases of dengue were reported with 6 deaths until September 14th this year (Roy, 2019). India has reported more than 67,000 cases of dengue fever as of October 13th (Dengue Fever, 2019). As of March 2020, 92 cases of dengue were found (Geographical distribution of dengue cases reported worldwide, 2020).

In the year of 2018, Sri Lanka observed 48,000 cases of dengue fever with 50 deaths (Gulf news, 2019). However, according to WHO, the number of dengue cases in the country this year till June was 25,216 (“Preventing Dengue in Sri Lanka”, 2019). According to the Epidemiology Unit of Ministry of Health, in 2018, 51,659 and 45,582 suspected dengue cases were observed until September 2019 (Dengue update, 2019). The districts that are under high risk of dengue this year include Colombo, Gampaha, Galle, Kalutara and Ratnapura as reported by the government deaths (Gulf news, 2019). As of July 2020, 23,217 cases were reported in the country (Geographical distribution of dengue cases reported worldwide, 2020). Thailand has declared a dengue hemorrhagic fever epidemic in 2019. According to Bangkok Post, on 2019, 28,785 dengue patients with 43 fatalities were reported (Wipatayotin, 2019). The incidence rate for this year was 61 people per 100,000, which was higher than that of last year. In 2018, the incidence rate was 38.6 people per 100,000 (Song, 2019). Until July 2020, 19, 758 cases were recorded in Thailand (Geographical distribution of dengue cases reported worldwide, 2020).

Due to the increasing prevalence of death in Bangladesh, Philippines, and other Asian countries by dengue, the unavailability of effective treatment in modern medicine and the prospective role of Papaya leaves from its traditional use in managing dengue fever, the current review was aimed at exploring and analyzing its therapeutic potential and safety/risk based on Pharmacological, Toxicological, and Clinical study reports. Besides, this study presents the etiology and pathogenesis of dengue prior to the mechanism of action, pharmacological, and clinical effectiveness of Papaya leaves to better understand it.

## Thrombocytopenia in Dengue

Dengue, which is transmitted by *Aedes aegypti*, is known as a Flaviviridae virus and has four serotypes numbered as Dengue Virus-1, Dengue Virus-2, Dengue Virus-3, and Dengue Virus-4 that stimulate the production of different antigens (Whitehorn and Farrar, 2010; Murphy and Whitehead, 2011). It is a single-stranded RNA virus that can be cleaved by host and proteases into three structural (C, capsid; prM, pre membrane; and E, envelope) and seven nonstructural (NS1, NS2a, NS2b, NS3, NS4A, NS4B, and NS5) proteins (Kurane et al., 1995). Structural protein E is associated with viral attachment, membrane fusion and virion assembly, whereas the nonstructural proteins are responsible for viral translation, transcription and replication (Crill, and Chang, 2004; Weaver and Vasilakis, 2009). The particular mechanism behind the dengue virus orchestrating low platelet count is not yet specified. However, different activities mediated by this virus in the host system are suggested to be likely responsible for thrombocytopenia as described below.

### Bone Marrow Suppression

**FIGURE 2:**
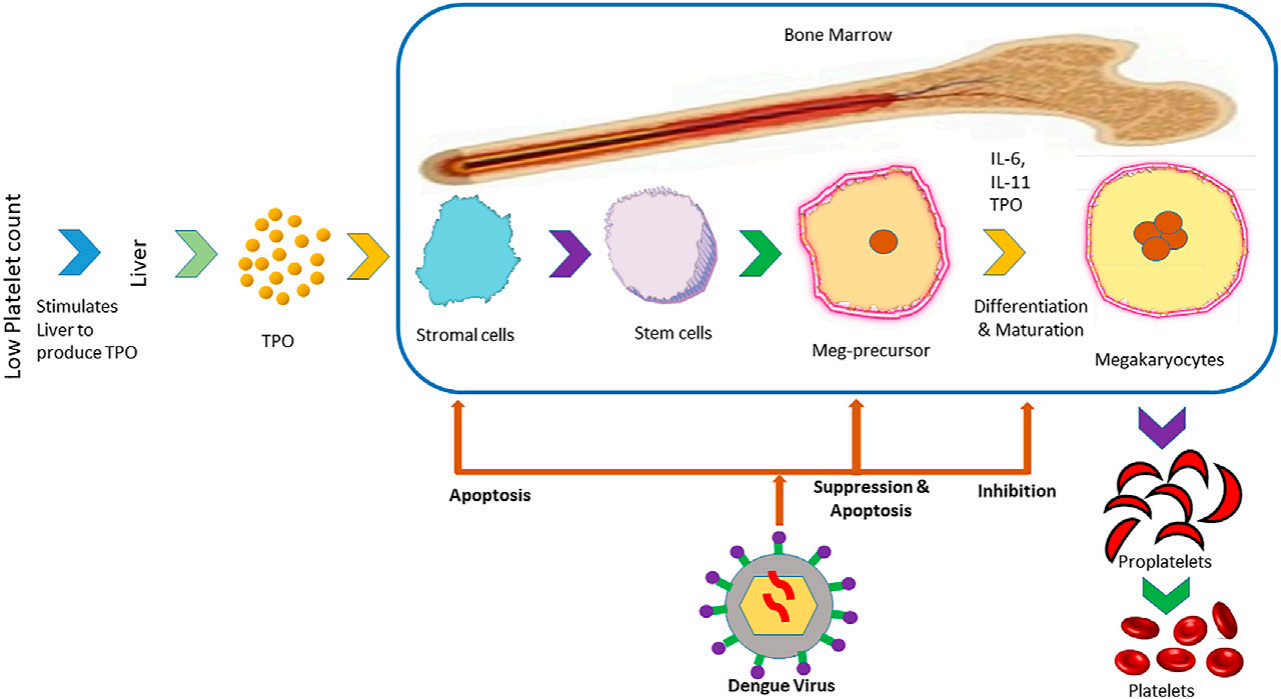
Mechanism of inhibition of the formation of platelets in bone marrow by dengue virus. The panel illustrates the steps in the formation of platelets from the liver that is stimulated by low platelet count in the blood. After the infection of the host with dengue virus, it prevents the formation of platelets from stromal cells and maturation in different stages.

The isolation of viral RNA from bone marrow of dengue infected individuals as well as hypocellularity in bone marrow and inhibition of maturation of megakaryocyte (Figure 2) during the primary stage of the disease suggest the suppressive effect of the dengue virus on bone marrow to cause thrombocytopenia (Kho et al., 1972; Srichaikul and Nimmannitya, 2000; de Araújo et al., 2009). DENV can exert this suppressive effect directly by damaging progenitor and stromal cells or indirectly by modifying cytokines produced by the bone marrow that are able to influence megakaryocyte differentiation (Srichaikul and Nimmannitya, 2000). Megakaryopoiesis is the formation process of megakaryocytes and development of platelets from them (Wahed and Dasgupta, 2015). The suppression of megakaryopoiesis of progenitor cells in mice inoculated with DENV-envelope protein domain III (DENV-EIII) has been observed *in vivo*. Similarly, the suppression of megakaryopoiesis of progenitor cells from murine bone marrow and human cord blood has been observed *in vitro*. Autophagy impairment and autophagy have been suggested as a possible mechanism behind the suppression of megakaryopoiesis (Lin et al., 2017). In another study, the propagation of DV-4 into human bone marrow progenitors that altered their proliferating potential has been observed (Murgue et al., 1997). It was discovered by Basu et al. (2008) that dengue virus inhibited the propagation of early megakaryopoietic progenitors by infecting and causing apoptotic cell death besides inhibiting the differentiation of CD34^+^ progenitors into megakaryocytes (Basu et al., 2008).

Thrombopoietin (TPO) is a cytokine that is responsible for regulating the production of megakaryocytes. TPO cytokines are produced in the response of low platelets, which bind with the TPO receptor, activate JAK and STAT pathways as well as stimulate the production of megakaryocytes (Kuter, 2013). TPO levels have been found to increase in the early stages of dengue, which suggest the stage of decreased megakaryopoiesis in that stage of disease (Matondang et al., 2004). DENV antigens have been found in DENV inoculated stromal cells (Rothwell et al., 1996). This infection of stromal cells presumably changes cytokine profiles such as in the case of TGF-β. This cytokine was found to reduce the production of pro-platelets apart from existing at a high level in dengue patients (Klinger, and Jelkmann, 2002; Badalucco et al., 2013).

### Destruction of Platelets

**FIGURE 3:**
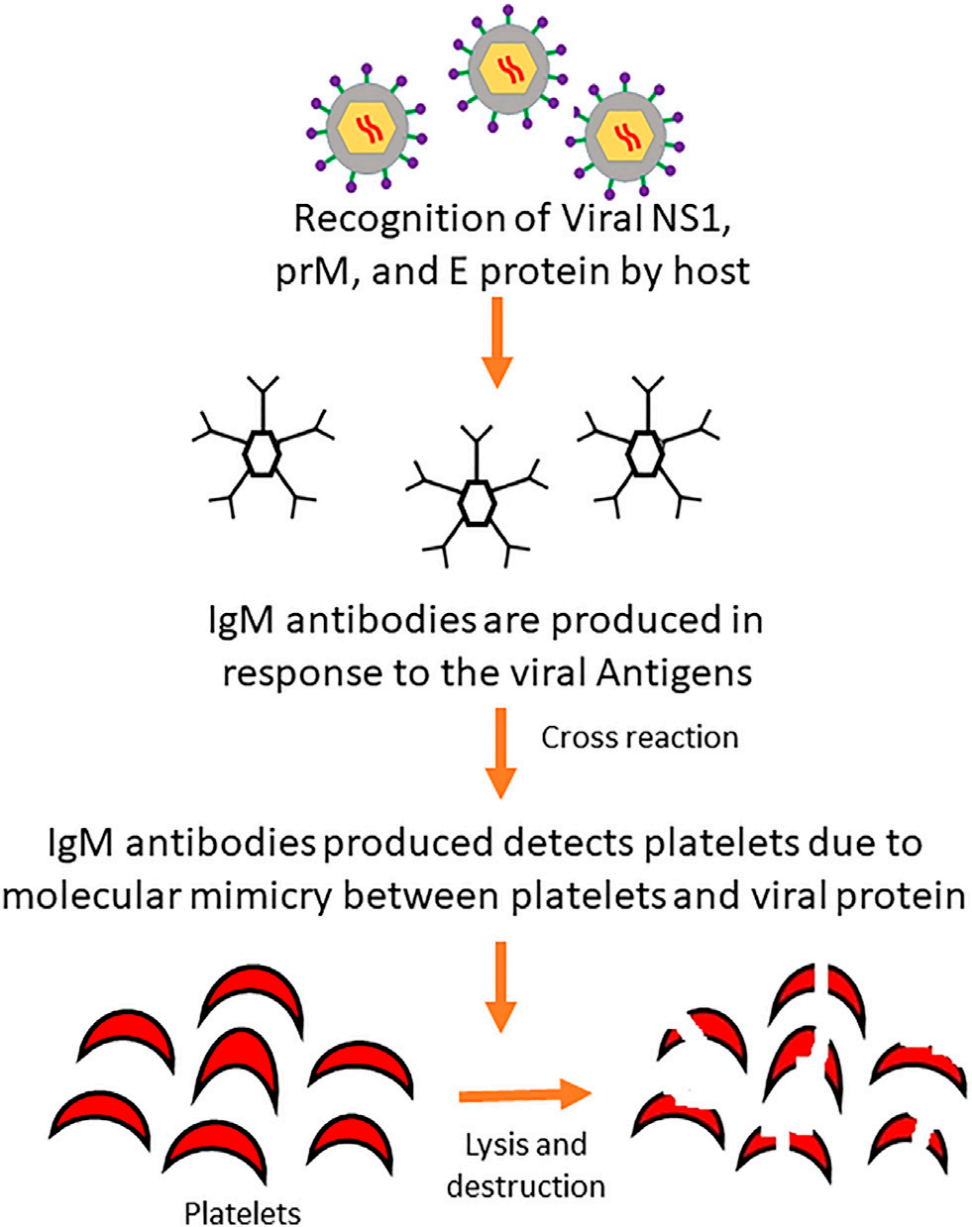
Mechanism of platelet destruction by antibody cross-reaction induced by dengue virus. Invading viral proteins mimics the platelets of the body and are destroyed by IgM antibodies produced upon recognition of viral proteins by the host defense system.

Molecular mimicry between viral proteins such as NS1, prM, and E with host platelet, endothelial cells and blood clotting molecules play a key role in the direct destruction of platelets (Figure 3) through cross-reactivity of antibodies or indirectly by producing aggregates with leukocytes or endothelial cells (Antiplatelet Trialists’ Collaboration, 1994; Hottz et al., 2011). Cross-reactivity of antibodies produced against these viral proteins can result in the activation of macrophage, platelet dysfunction, coagulation deficiencies and destruction of endothelial cells (Lin et al., 2011) Furthermore, the IgM class of antibodies produced against NS1 viral proteins are capable of inducing peripheral platelet destruction or cell lysis and inhibiting platelet aggregation. These IgM antibodies have been found in a high amount in patients with Dengue Hemorrhagic Fever (DHF) than those with Dengue Fever (DF) as well as increased platelet lysis in DHF patients (Lin et al., 2001; Wan et al., 2013).

**FIGURE 4:**
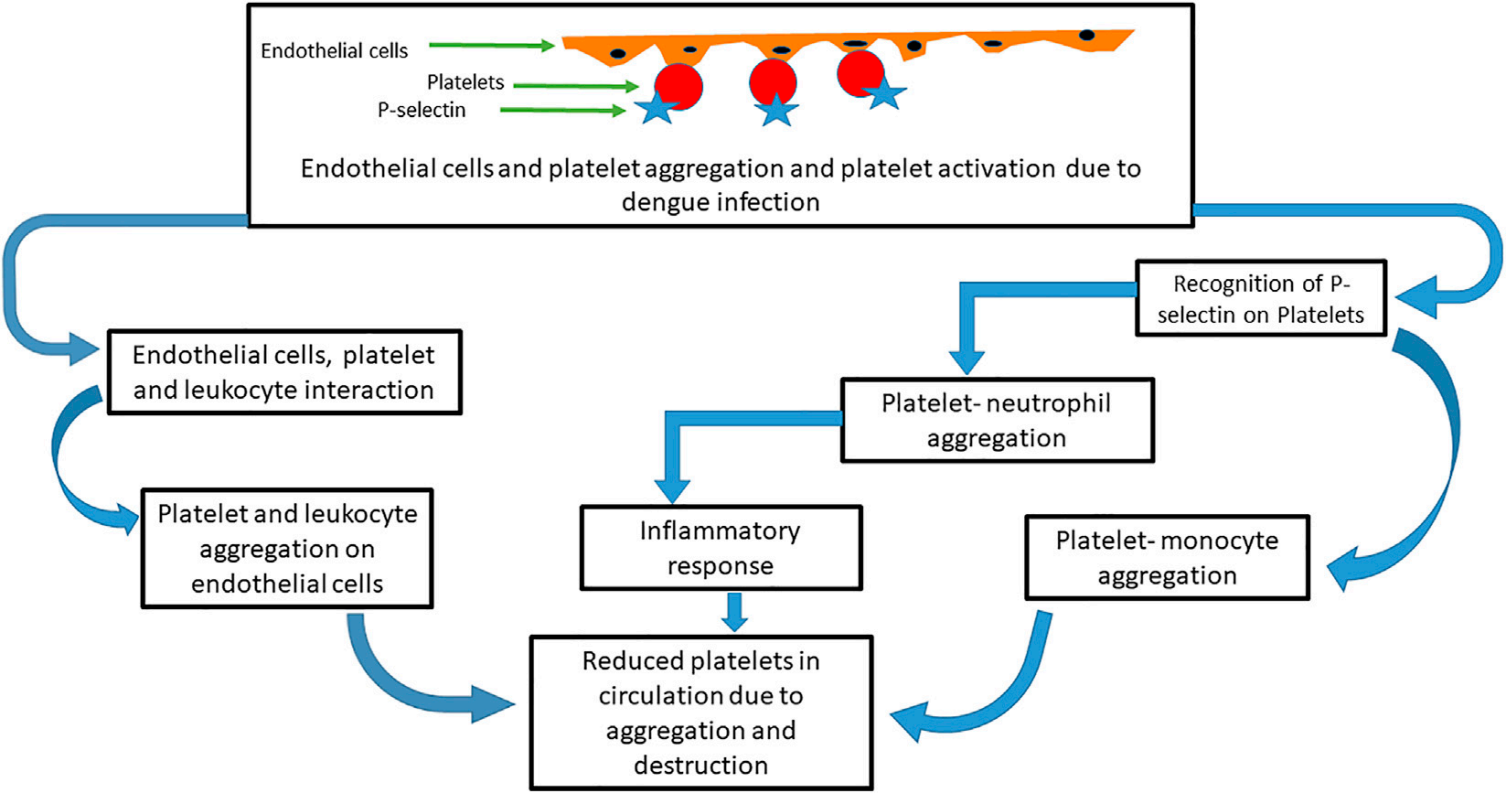
Mechanism of platelets and other cells’ aggregation in thrombocytopenia induced by dengue virus. After dengue infection, platelets are aggregated with endothelial cells that in turn activate the platelets. P-selection proteins on platelet cells are recognized by neutrophils and monocytes and form further aggregation. Leukocytes also interact with the platelet endothelial cell aggregates. These aggregations reduce the available platelet count and induce an inflammatory response that destroys the platelets.

In addition, dengue infection causes endothelial cells to activate, which in turn causes platelet adhesion and activation as well as express P-selectin on its surfaces (Krishnamurti et al., 2002; Sosothikul et al., 2007; Dalrymple and Mackow, 2011; Lin et al., 2011). The expression of P-selectin results in the interaction of activated platelets with leukocytes. Platelet-monocyte and platelet-neutrophil aggregates are also formed from the expression of P-selectin (Onlamoon et al., 2010). Aggregation of platelets of endothelial cells has been observed to cause thrombocytopenia (Butthep et al., 1993). The formation of this aggregation (Figure 4) contributes to thrombocytopenia by cell death or reduction in the number of available circulating platelets (Andrews et al., 2014).

### Dysfunction of Platelets

Platelet dysfunction has been reported in many investigations. DV has been reported to activate platelets and suppress platelet aggregation (Lin et al., 2001; Sun et al., 2007). Platelet activation related to morphological changes in platelets such as degranulation, alteration in platelet membrane structure, presence of filopodia, and dilatation of the open canalicular system have been observed in the case of dengue virus inoculated platelets (Ghosh et al., 2008). NS1 of DENV was also found to activate platelets by binding with Toll-like receptor four on platelets (Chao et al., 2019). An increase in secretion of beta-thromboglobulin (BTG) and platelet factor 4 (PF4) related to the suppression of platelet aggregation has been observed (Srichaikul et al., 1989). Antibodies produced against NS1 viral proteins recognize protein disulfide isomerase (PDI) on the surface of platelets, inhibiting them and the aggregation of platelets partially due to the inhibition of PDIs by those antibodies (Chen et al., 2009; Cheng et al., 2009). Increased L-arginine transport and nitric oxide generation in platelets of dengue patients have also been regarded as underlying mechanisms behind reduced platelet aggregation (Mendes-Ribeiro et al., 2008).

Several activities of platelets are influenced by a dengue infection. Anti-inflammatory properties have been observed to be exerted by the activation of platelets due to dengue infection. Activation of platelets causes the interaction of CD40 and CD40L, leading to an increase production of IL-10 and suppression of TNF-α by monocytes (Gudbrandsdottir et al., 2013). IL-10 has also been found to be associated with low platelet counts (Azeredo et al., 2001). Platelets are a source of TGF-β1 and in dengue infected patients, the levels of TGF-β1 were found to be low (Assoian et al., 1983; Djamiatun et al., 2011).

### Role of Blood Coagulation Factors

Disseminated intravascular coagulation (DIC) is activated during dengue infection as DENV activates fibrinolysis and coagulation as well as changes in coagulation and fibrinolytic parameters (da Costa Barros and de-Oliveira-Pinto, 2018). It has been observed that low platelet counts are associated with prothrombin, fibrinogen, factor VIII, plasminogen, antithrombin activities, and prolongation of partial thromboplastin time (PTT) and prothrombin time (PT) in DHF patients with DIC (Funahara et al., 1987). Elevated activation of platelets, fibrin formation, and thrombus deposition occur in blood circulation due to initiation of DIC that can lead to organ failure, while the expenditure of platelets and coagulation factors by DIC can result in hemorrhagic disturbances (Azeredo et al., 2015).

### Platelets as a Target for Viral Replication

**FIGURE 5:**
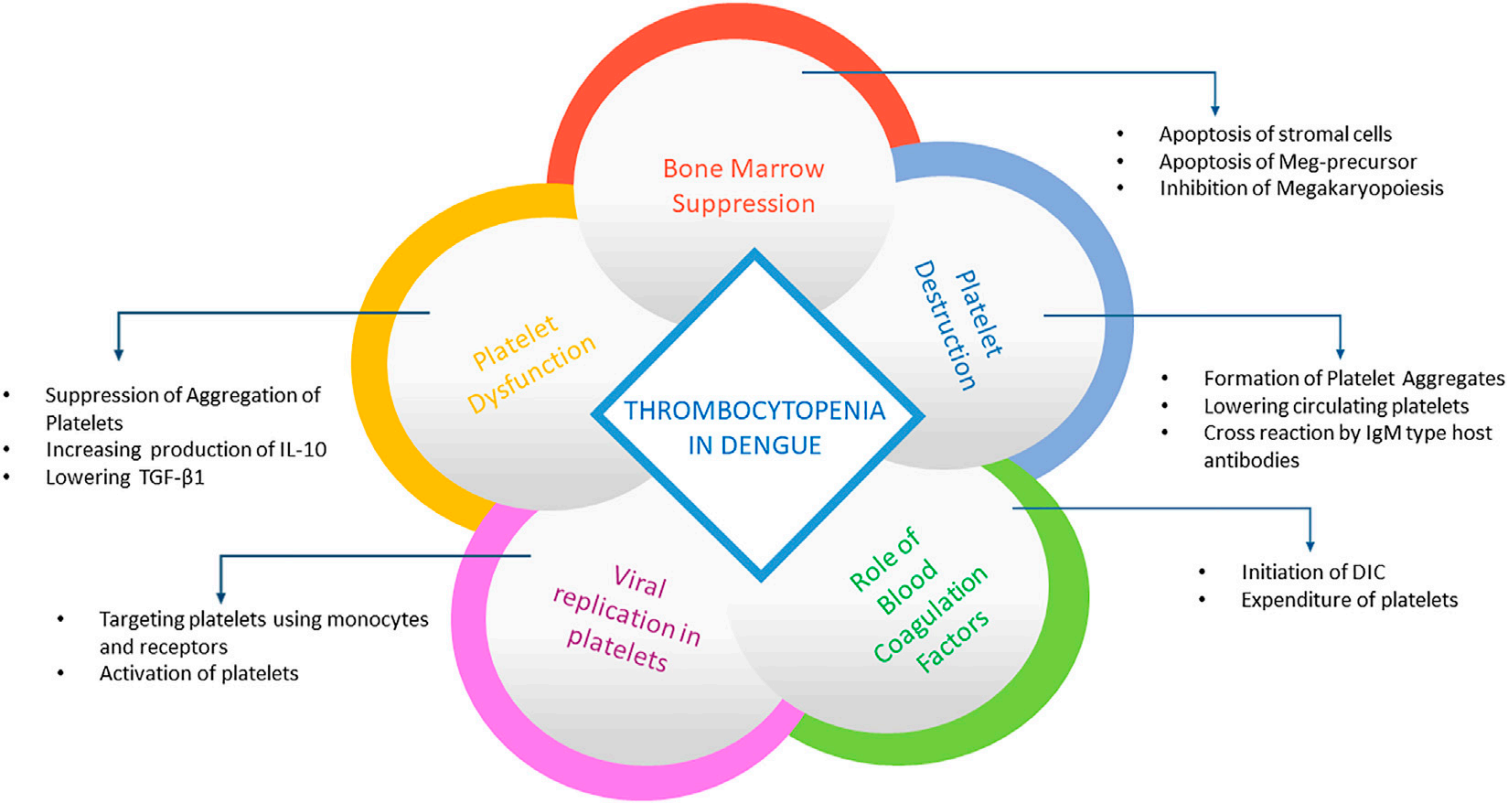
Summary of processes behind thrombocytopenia in dengue. The panel summarizes how platelets are destroyed after dengue infection. After viral replication in platelets, it activates platelets using receptors and monocytes as well as aggregates the platelets, reducing its availability. It also suppresses bone marrow and prevents platelet formation by destroying premature cells.

It has been observed that DENV is associated with platelet activation and replicates in them, which suggests that platelet clearance may be mediated by DENV exposed platelets with CD14^+^CD16^+^ monocyte even if DENV does not cause platelet infection directly (Kar et al., 2017). However, intact platelets have been found to replicate all four serotypes of dengue (Rondina and Weyrich, 2015). Also, platelets have been discovered to replicate with the virus both at 37° and 25° as well as the saturable binding of platelets with DENV, thus producing an infectious virus (Simon et al., 2015). DENV RNA has been found in platelets like CD61^+^ cells in an investigation using blood from dengue patients and experimentally infected rhesus monkey. In addition, the presence of DENV antigens has been found in the vesicle of different sizes and in nuclear cells like platelets (Noisakran et al., 2012). Moreover, DENV-like particles, DENV antigens, and DENV RNA have been found with platelets in other studies (Noisakran et al., 2009b; Noisakran et al., 2009a). DENV has been suggested to cause activation, mitochondrial dysfunction, and platelet apoptosis by targeting Dendritic cell-specific intercellular adhesion molecule-3-grabbing nonintegrin (DC-SIGN) receptors on platelets (Hottz et al., 2013; Hottz et al., 2018). A summary of the possible mechanism behind the induction of thrombocytopenia is illustrated in Figure 5.

## Chemical Composition of *Carica papaya* L. Leaves

**FIGURE 6:**
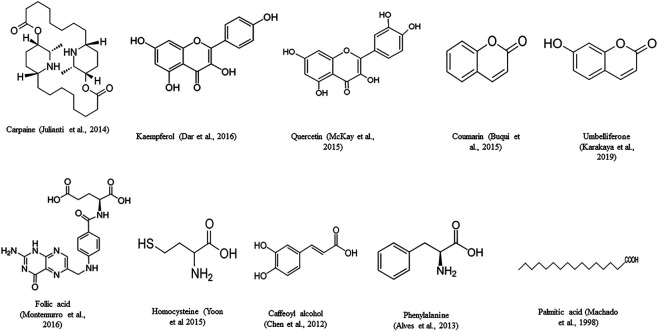
Phytocompounds isolated from *Carica papaya* L. leaves.

Leaves of papaya have been found to have multiple chemical compounds (Figure 6) namely alkaloids, terpenoids, phenols, tannins, flavonoids, saponins and glycosides (Ikeyi et al., 2013; Nath and Dutta, 2016; Singh et al., 2018). Papaya leaves have also been reported to constitute dry matter of 89.60%, total ash of 18.3%, crude fat of 3.5%, crude protein of 13.1%, crude fibre of 1.95%, nitrogen-free extract of 63.1%, acid insoluble ash of 4.4%, phosphorous of 0.41%, and calcium of 2.49% (Nath and Dutta, 2016). Carpaine was found to be present in the leaves (Head and Lauter, 1956; Teng et al., 2019). Teng et al. (2019) isolated carpaine from alkaloidal hexane extract of Papaya leaves and confirmed the identity of the compound “carpaine” by NMR, MS, FT-IR, and X-ray crystal data (Teng et al., 2019) Akhila and Vijayalakshmi (2015) have reported and identified 21 compounds present in the aqueous extracts of the leaves. They were tocopherol, ascorbic acid, carpaine, deoxykaempferol, kaempferol, deoxyquercetin, quercetin, dicoumarol, coumaroylquinic acid, coumarin, folic acid, cysteine, homocysteine, cysteine sulphoxide, l glutamic acid, p-coumaroyl alcohol, dimethoxy phenol, umbelliferone, phenylalanine, caffeoyl alcohol, and methyl nonyl ketone (Akhila and Vijayalakshmi, 2015; Dar et al., 2016; Karakaya et al., 2019). Another study revealed the presence of decylene, trans-geranylacetone, methyl tridecanoate, palmitic acid, myristic acid, hexadecanoic acid, linolelaidic acid, methyl cis-6-octadecenoate, stearic acid, oleic acid, 15-tetracosenoic acid, methyl heptacosanoate, trans-13-docosenoic acid, methyl erucate, methyl behenate, heneicosanoic acid, farnesyl cyanide in n-hexane, and methanol (60:40% ratios) extract of the leaves. In the study, minerals such as manganese, coper, cadmium, iron, cobalt, and zinc were also observed in the leave extract of papaya (Oche et al., 2017). Additionally, papain enzyme can also be extracted from papaya leaves (Welde and Worku, 2018).

## Pharmacological and Therapeutic Potentials of *C. papaya* Leaves in Dengue

### Anti-Dengue Activity of Papaya Leaves: Evidence From *in vitro* and *in vivo* Model Studies

Several *in vitro* investigations have reported an increase in platelet count after an administration of papaya leave extract. Zunjar et al. (2016) evaluated the antithrombocytopenic activity of alkaloid extract of Carica papaya L. leaves and its isolated bioactive compound “carpaine” in busulfan induced thrombocytopenic Wistar rats. The study found that carpaie potentially sustained platelet counts up to 555.50 ± 85.17 × 10^9^ cells/L without showing acute toxicity in rats. The authors concluded that the main phytocompound(s) responsible for antithrombocytopenic activity of *C. papaya* L. leaves are alkaloids, particularly the “carpaine” alkaloid, rather than phenolic compounds (Zunjar et al., 2016). Administration of aqueous extract of papaya leave extract in DENV-2 infected Vero cell from the African green monkey kidney cells (ATCC No. CCL-81) resulted in a reduction in foci formation. Papaya leave extract demonstrated its potential against intracellular replication of DENV-2. IC50 value, which was observed to be 137.6 μgmL-1 for 5h before the infection and four days after the infection. The CC50 and selective index values were found to be 10,437 (μgmL-1) and 75.85, respectively (Salim and Abu, 2018). Significant reduction in expression of the envelope (*p* < 0.001) and NS1 proteins (*p* < 0.001) was observed in DENV-infected THP-1 cells after aqueous extract of the plant leaves was administered. In the study, IFN-α expression was reported to be 1.6-fold higher in DENV-infected THP-1-cells treated with the leave extract. Significant reduction in erythrocyte damage and hydrogen-peroxide-induced lipid peroxidation (*p* < 0.001) has been also observed after treatment with papaya leave extract (Sharma et al., 2019). In another study, it was observed that the chloroform extract of the leaves showed inhibitory activity (EC50 => 1 mgml−1) against DENV2 with a selectivity index value of ± >1 when Anti DENV2 activity was investigated on LLC-MK2 cell line (Adarsh et al., 2017).

Treatment of dengue infected AG129 mice with papaya leaf juice for three consecutive days after 24h of dengue virus inoculation resulted in increased plasma CCL2/MCP-1 level during the peak of viremia (Norahmad et al., 2019). No effect on the expression of plasma NS1and dengue viral RNA levels was seen in dengue infected AG129 mice after being treated with oral treatment of 500 mg/kg/day and 1,000 mg/kg/day of freeze-dried papaya leave juice. However, morbidity level was seen to decrease in the infected rats (Razak et al., 2018). In an *in vitro* study, a significant reduction (*p* < 0.05) in platelet aggregation has been observed in platelet-poor plasma collected from dengue patients after treatment with papaya leaves (Chinnappan et al., 2016). Carpaine, isolated from alkaloidal hexane extract of *Carica papaya* L. leaves, has been reported to possess antiplasmodial activity *in vitro* and *in vivo* (Jilanti et al., 2014; Teng et al., 2019). Carpaine exhibited potential antimalarial activity against both of the *P. falciparum* 3D7(IC_50_ 2.01 ± 0.18 μg/ml) and Dd2 strains (IC_50_ 2.19 ± 0.60 μg/ml) (Teng et al., 2019) due to a direct inhibitory action against the parasite. Teng et al. (2019) also reported that carpaine exhibits antimalarial activity by displaying a high selectivity to the malarial parasite and is non-toxic to normal human red blood cells.

### Larvicidal Activities of *Carica papaya* L. Leaves Against *Aedes aegypti*

In a study using aqueous and ethanol extract of leaf, bark, root, and seed of papaya against larvae of *Aedes aegypti*, it was found that the 2^nd^ and 4^th^ stage mosquito larvae had 83 and 86% mortality, respectively, against aqueous extract of papaya seeds. Meanwhile, the mortality percentage of 2^nd^ and 4^th^ stage larvae was 96 and 100%, respectively, against ethanolic extract of the seeds (Malathi and Vasugi, 2015). Another investigation reported the LC50 and LC90 of the papaya leave juice to instar I larvae, which were 4.1 and 15.5%, respectively. In the case of instar III larvae, LC50 and LC90 were 10.6 and 18.3%, respectively (Cahyati et al., 2019). The chloroform extract of *Carica papaya* L. latex was found to have better larvicidal activity when compared with methanol and aqueous extracts (mortality rate of *A. aegypti* II instar 90.33% and III instar 98.33%) (Chandrasekaran et al., 2018). The mortality rate of *A. aegypti* has been reported in another investigation to be 100% against the aqueous extract of the seed at the concentration of 250 and 500 mg/L. In the study, the mortality rate of the larvae was found to be 100% at the concentration of 500 mg/L of papaya peel (Hayatie et al., 2015).

### Immunomodulatory Activities of *Carica papaya* L*.* Leaves

Saponins isolated from papaya leaves have been considered to increase cell mediated immunity as well as humoral antibody by stimulating increased production of antibodies in animal models (Gupta et al., 2017; Ojiako et al., 2019). In an *in vitro* study model, aqueous extract of papaya leaves have been found to reduce the production of IL-2 and IL-4 and enhance the production of IL-12p40, IL-12p70, IFN-γ, and TNF-α (Otsuki et al., 2010). Furthermore, in another *in vivo* investigation, papaya leaves extract were found to reduce the expression of IL-4, IL-5, eotaxin, TNF-α, NF-ĸB, and iNOS in mice with ovalbumin- (OVA) induced allergic asthma (Inam et al., 2017). Ethanolic extract of papaya leaves was found to significantly inhibit (*p* < 0.05) isopentenyl pyrophosphate induced TNF-α production in LPS-induced dendritic cells (Sagnia et al., 2014). The inhibition of release of pro-inflammatory TNFa, IL-1a, IL-1b, IL-6 and IL-8 has been also observed by the methanol extract of papaya leaves (Salim et al., 2014).

### Papaya Leaves to Improve Thrombocytopenia in Dengue Patients

Dengue patients have been reported to have elevated platelet count after administration with papaya leaves in multiple investigations. In a study conducted on 300 dengue patients across five health centers, a significant increase (*p* < 0.01) in platelet count has been observed within five days of therapy sessions, for three times a day, in the intervention group treated with the papaya leave extract tablets (1,100 mg) for three times a day (Kasture et al., 2016). However, the effectiveness of the 1,100 mg tablet three times a day for long term treatment has not been mentioned. Another study has been conducted by the Department of Medicine, S.P. Medical College and Associated Group of P.B.M. Hospitals, Bikaner on 400 patients (275 males and 125 females) with dengue fever and associated thrombocytopenia. The study group was given papaya leaf extract capsule (500 mg) once daily along with routine supportive treatment (antipyretic Paracetamol, intravenous 0.9% normal saline, antiemetic) for five consecutive days. From the third day of the treatment, significantly increased (*p* < 0.01) platelet count was observed (Gadhwal et al., 2016). A significant increase (*p* < 0.01) in platelet count has been also reported in a study where 30 patients with dengue fever associated thrombocytopenia were given *Carica papaya L.* leaf extract (CPLE) (Gowda et al., 2015). Venugopal (2018) reported an early increase in platelet count and reduced average duration of hospital stay among the patients treated with papaya leaves extract when compared with those of the control group. The average duration of hospital stay was 5.42 days for patients treated with the extract and 7.2 days for the control group. Furthermore, platelet requirement by blood transfusion was more in the control group than that in the treated group. The study included a total of 500 patients (380 males, 120 females) where the treated group was given papaya leaf extract (CPLE) in the dose of 1,100 mg three times daily for five days along with symptomatic and supportive treatment. The control group was given symptomatic and supportive treatment only (Venugopal, 2018). In an investigation on 228 patients with dengue hemorrhagic fever, half of the patients were included in the treated group and were administered orally with papaya leaves juice for three days. Their blood parameters were monitored every 8h. A significant increase (*p* < 0.001) in platelet count was observed in the group treated with the juice (Subenthiran et al., 2013).

A 23 year-old male dengue associated thrombocytopenic patient has been reported to have increased platelet count with every administration of the extract. Platelet count increased from 28000/micro liter to 138000/micro liter by the end of the treatment duration of five days (Siddique et al., 2014). In another case, a 45year-old dengue patient who received 25 ml of aqueous extract of papaya leaves twice daily for five consecutive days showed an increased platelet count from 55 × 10 (3)/µL to 168 × 10 (3)/µL, white blood cells from 3.7 × 10 (3)/µL to 7.7 × 10 (3)/µL and neutrophils from 46.0 to 78.3% (Nisar et al., 2011).

A systemic review and meta-analysis on clinical reports of papaya leaves in dengue have been conducted by Rajapakse et al. (2019) where 86 studies were screened and nine were selected based on the inclusion criteria. Among these selected studies, seven of them reported the anti-thrombocytopenic potential of papaya leaves extract. In the study, it was found that the extract had reduced the duration of hospital stay (mean difference −1.98 days, 95% confidence interval −1.83 to −2.12, three studies, 580 participants, low-quality evidence) and improved mean platelet counts between the first and fifth day of treatment (mean difference of 35.45, 95% confidence interval from 23.74 to 47.15, three studies, 129 participants, low-quality evidence) (Rajapakse et al., 2019). In another systemic interview and meta-analysis that analyzed data of 377 subjects, it was found that papaya leaf extract was associated with elevation of platelet counts in the overall study (mean difference [MD] = 20.27 [95% confidence interval (CI) 6.21–34.73; *p* = 0.005]). These extracts were also found to decrease the duration of hospital stay (MD = 1.90 [95% CI 1.62–2.18; *p* < 0.00001]) (Charan et al., 2016).

Shetty et al. (2019) reported an increase in platelet count (*p* = 0.15) in children aged one to 16 years old with dengue and platelet counts from 1.5 × 105 per μL to 50 × 103 per μL. In the study, 30 children were divided into control and test groups where the test group received 1,100 mg tablet (>12 years), syrup of (275 mg/5ml) 10 ml (between 6 and 12 years) and 5ml for (<6years), which were given thrice daily for five days along with routine symptomatic treatments (Shetty et al., 2019). A significant increase (*p* < 0.01) in platelet count has been reported in a study where each dengue patient of 100 cases was either in the study or the control group. The study group was treated with papaya leaf extract at the dose of 500 mg thrice a day while the control group received placebo capsules in the same frequency for five consecutive days (Adarsh et al., 2017). In an investigation conducted on nine dengue patients with decreased platelet count admitted into Vishnu Sri Hospital, Tirupati, Andhra Pradesh, who were given 5ml of papaya leaf extract three times a day at 6h intervals for five days were found to have a significant increase (*p* < 0.05) in WBC and platelet counts (Naresh et al., 2015). Similarly, platelet count was found to be increasing faster in the group treated with papaya leaves extract at the dose of 10ml at 8h intervals for five days when compared with the control group. In the study, 80 dengue patients were randomized into two groups; study group and control group (Sharma and Sharma, 2017). Papaya leaves juice was reported to increase platelet count within 24h after drinking two tablespoons of the juice every 6h in the case of five dengue patients with low platelet counts in the blood (<150,000) (Kala, 2012). In a study on 39 dengue patients with platelet counts ≤50 × 109/L, an increased trend in platelet count was observed in the treated group who were given 5ml of leaves extract of papaya twice a day for four days (Assir et al., 2011). In a study where 80 dengue patients were randomized into control and intervention groups, the intervention group was given two papaya leaves capsules three times daily. It was found that the intervention group has significantly elevated platelet count and reduced hospitalization time (*p* < 0.05, *p* < 0.05, respectively) (Yunita et al., 2012). In a study on 294 dengue patients (aged between one and 12) with thrombocytopenia that were randomized into a control and intervention group, the platelet count intervention group was found to be significantly increased when compared to the control group after being administrated with papaya leaf syrup (*p* < 0.05) (Srikanth et al., 2019).

### Possible Mechanisms of Actions of Papaya Leaves to Improve Thrombocytopenia

**FIGURE 7:**
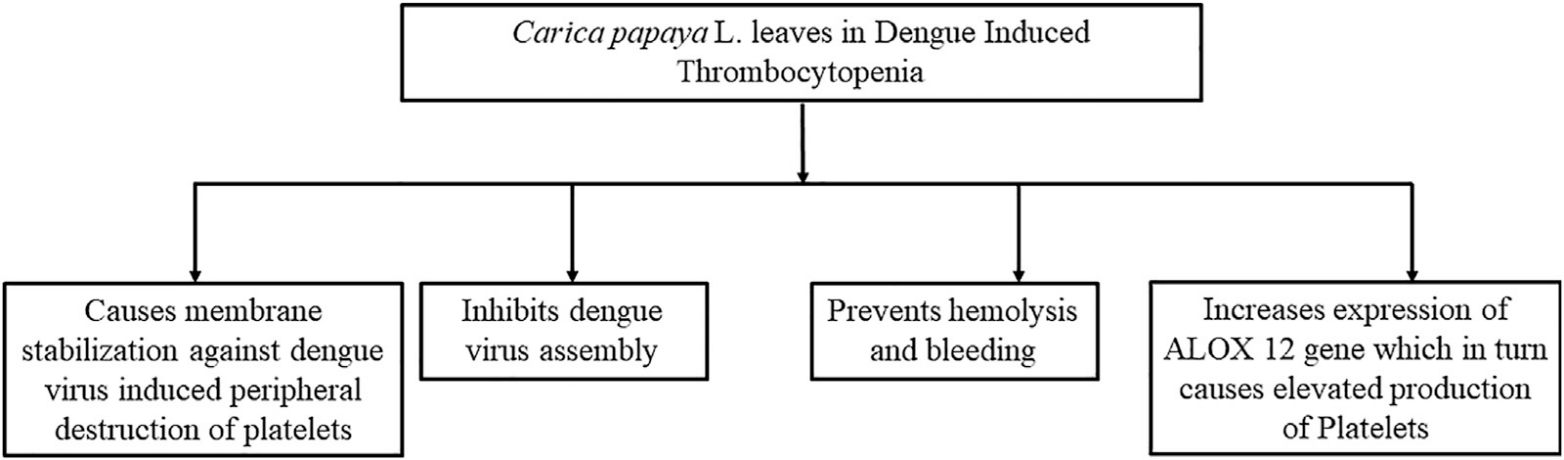
Possible mechanisms behind the role of papaya leaves in improving platelet count. This figure summarizes the mechanism of papaya leaves in reducing thrombocytopenia by the dengue virus. It stabilizes the membrane of platelets and reduces platelet destruction. It also prevents viral assembly in cells and hemolysis. Furthermore, it increases the expression of genes that elevate the production of platelets.

**FIGURE 8:**
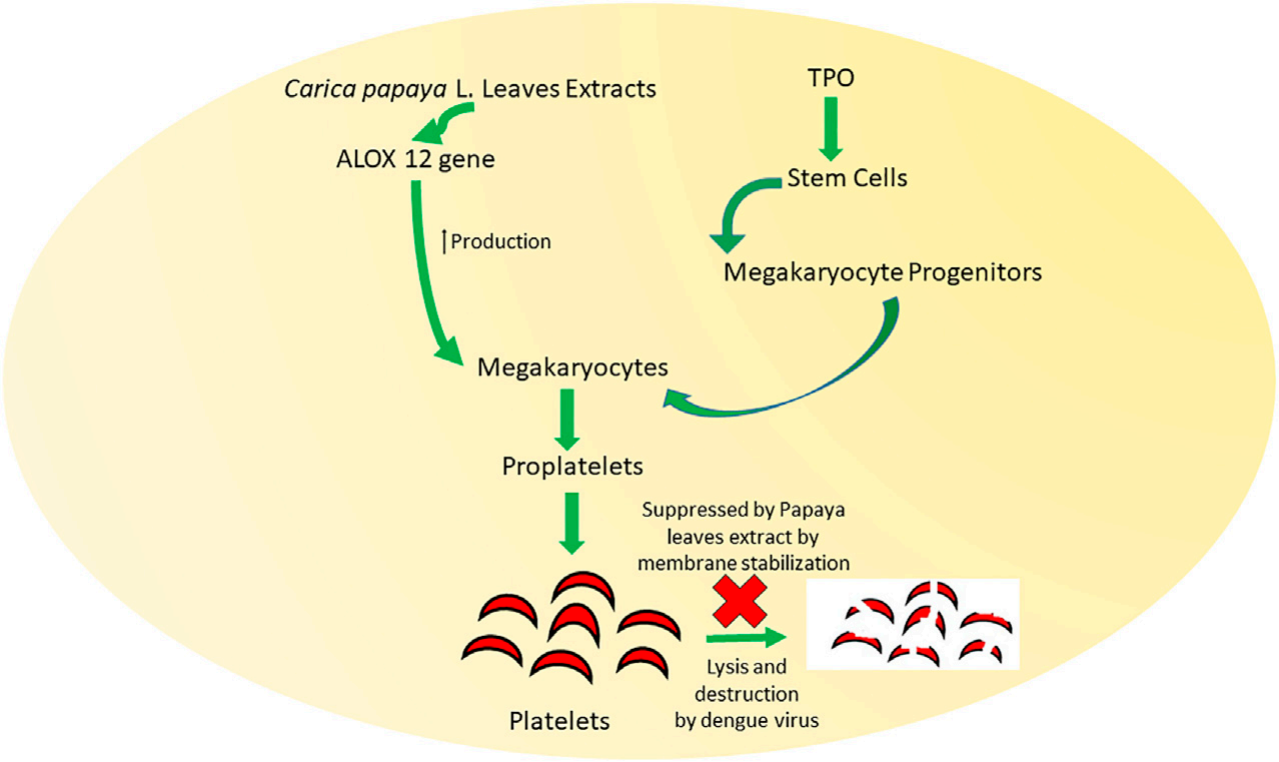
Mechanisms behind the role of papaya leaves in improving platelet count by membrane stabilization and production of ALOX 12 gene. *Carica papaya* L. leaf extract promotes the formation of megakaryocytes and megakaryocyte progenitors that induce the formation of proplatelets. Lysis and destruction of these proplatelets are also inhibited by *Carica papaya* L. leave extract.

Several mechanisms (Figure 7) have been suggested to explain the potential of papaya leaves to improve platelet counts. Flavonoids isolated from papaya leaves have been reported to inhibit a protease involved in viral assembly (Charan et al., 2016). It has been proposed that dengue virus serotype two can bind to platelets directly to destroy them or can cause their peripheral destruction indirectly by promoting the production of anti-platelet or cross-reaction antibodies (da Costa Barros and de-Oliveira-Pinto, 2018). Papaya leaf extract has been reported to cause membrane stabilization, which may facilitate the reversal of peripheral platelet destruction by dengue virus (Ranasinghe et al., 2012). The possible role of antioxidant and free radical scavenging properties of papaya leaf extract in aiding the prevention of hemolysis and bleeding have also been suggested (Pandita et al., 2019). However, dengue virus induces the reduction in proliferation of platelets by inhibiting megakaryocytopoiesis or inhibiting differentiation of stem cells into megakaryocyte precursor cells (da Costa Barros and de-Oliveira-Pinto, 2018). Papaya leave extract has been found to increase expression ALOX 12 (Figure 8) gene by 15-fold, which further increases megakaryocyte production and its conversion into platelets as well as the production of platelets through the 12-HETE mediated pathway (McRedmond et al., 2004; Sundarmurthy et al., 2017).

Quercetin isolated from *Carica papaya* L. leaves has been found to inhibit NS2B-NS3 serine protease, which is essential for dengue viral assembly (McKay et al., 2015; Senthilvel et al., 2013). Quercitin has been also found to suppress platelet aggregation induced by ADP (Tzeng et al., 1991). In an *in vitro* study, myristic acid has been discovered to inhibit thrombin-induced aggregation of rabbit platelets (Fukamachi et al., 1988). Coumarin was found to inhibit e DEN2 NS2B/NS3 protease present in dengue virus in a molecular docking study (Rathnayake et al., 2020). Furthermore, the inhibition of platelet aggregation and prevention on thrombosis by the blocking of enzymatic activities of thrombin and FXa by kaempferol that is also found in papaya leaves have been reported on ICR (Imprinting Control Region) mice and SD (Sprague Dawley) rats (Choi et al., 2015). Anti-thrombocytopenic activity of alkaloid extract and carpaine has been observed in busulfan induced thrombocytopenic Wistar rats (Zunjar et al., 2016). Moreover, papain isolated from papaya leaves has been also found to reverse immune-mediated platelet destruction (Lavanya et al., 2018).

### Toxicity Study Reports of *Carica papaya* L. Leaves

In an investigation where acute toxicity and sub-chronic toxicity of hydroalcoholic extracts of papaya leaves in chicks were observed, no biochemical or hematological changes were reported. In the investigation, papaya leaves extract was given at doses ranging from 40 to 5,120 mg/kg for acute toxicity study and 80, 160, 320, and 640 mg/kg/day for 42 days for sub-acute study (Nghonjuyi et al., 2016). Another investigation has been carried out using methanolic extract on mice acute toxicity and sub-chronic toxicity. For acute toxicity study, doses at 200 mg, 400 mg, 800 mg, 1,600 mg, and 3,200 mg/kg for 24h were given, while for sub-chronic study, similar doses for 60 days were administered. The papaya leaves extract did not show any toxicological changes (Peristiowati and Puspitasari, 2019). In another study where acute toxicity and LC50 values of alcoholic extract of brown dried pawpaw (*Carica papaya* L.) leaf on fingerlings of African catfish *CLarias gariepinus* was observed, behavioral changes such as air gulping, erratic swimming, discoloration, loss of reflex, and skin peeling as well as a decrease in packed cells volume (PCV), hemoglobin (Hb), red blood cell (RBC), mean corpuscular haemoglobin concentration (MCHC), and an increase in the mean corpuscular hemoglobin (MCH) and mean corpuscular volume (MCV) were recorded in a time-and dose-dependent manner. The extract was given for four days at doses of 4.40, 8.80, 13.20, 17.60, and 22.00 ml/L and compared with the control group. In terms of behavioral change, gasping occurred in all five doses and other behavioral changes occurred in doses ranging from 13.20 to 22.0 ml/L. Considerable hematological changes occurred especially at the doses of 13.20, 17.60, and 22.00 ml/L when compared with control. LC50 value was found to be at a concentration of 10.9 ml/L (Ishaku et al., 2019). In an *in vivo* study, papaya leave extract at concentrations of 5, 50, 300, and 2000 mg/kg was investigated in Sprague Dawley rats for toxicity. No toxicological changes were found in the study of triglyceride using other biochemistry parameters. The weight of internal organs was also within a normal range. However, a significant increase (*p* < 0.05) in hemoglobin (HGB), hematocrit (HCT), red blood cell (RBC), and total protein suggesting dehydration was observed (Halim et al., 2011). However, in another sub-chronic investigation of papaya leaf extract given in concentrations of 0.01, 0.14, and 2 g/kg body weight (BW) for 13weeks on Sprague Dawley rats, no changes in hematological and biochemical parameters were found (Ismail et al., 2014).

A study has been conducted using the leaves of “Cavite Special” and “Sunrise Solo” varieties of papaya grown in the Philippines to examine the toxicity in Brine Shrimp. The LC50 values for “Cavite Special” and “Sunrise Solo” varieties were found to be 421 μg/ml and 132 μg/ml, respectively, which revealed that “Sunrise Solo” variety is less toxic than “Cavite Special” (Madjos and Luceño, 2019). In another study, methanolic extract of papaya leaves has been found to have anti-fertility effects as it caused a dose-dependent decrease in sperm count significantly (*p* < 0.05) in male Wister rats. In the study, normal sperm count and biochemical profile were observed at the dose of 100 mg/kg body weight (Nkeiruka and Chinaka, 2013). Another study reported that papaya leaf extract was found to be safe up to 2 g/kg body weight. In the study, papaya leaf extract was given to Sprague Dawley rats at doses of 0.01, 0.14, and 2 g/kg for 28 days (Afzan et al., 2012). In another study, the LC50 value of methanolic extract of the leaves was found to be 118.73 mg/ml (Hossain et al., 2019).

### Anti-Dengue Potential of Plants Other Than *Carica papaya* Leaves

Guava leaves have quercitin like papaya leaves, can improve platelet counts, and also have anti-dengue effects. It has been found to inhibit the formation of enzyme mRNA in the virus (Abd Kadir et al., 2013). *Andrographis paniculata* is a plant that contains diterpenoids, flavonoids, and polyphenols, and was found to have dengue virus inhibitory potential in Vero E6 cells (Chao and Lin, 2010; Abd Kadir et al., 2013). *Cladogynos orientalis* also contains quercitin, diterpins, and flavonoids and was found to have anti-dengue effects. In an MTT analysis, dichloromethane ethanol extract of was found to inhibit DENV-2 at a concentration of 12.5 μg ml−1 with 34.85% (Klawikkan et al., 2010; Sithisarn eta l., 2015). *Azadirachta indica* L. also contains quercitin and was found to have anti-dengue virus replication effects. The aqueous extract of neem leaves was observed to completely inhibit 100–10,000 tissue culture infective dose (TCID)50 of the virus (Parida et al., 2002; Alzohairy, 2016).

## Conclusion and Future Directions

Considering the four varieties of viruses responsible for dengue fever, one person can suffer from dengue multiple times although being infected once with a particular serotype provides long term immunity against that serotype. Dengue infection arrays a wide range of symptoms including fever, rash, extreme muscle pain, and headache as well as clinical manifestations such as multiple organ failures and thrombocytopenia, and an abnormal lowering of platelet count. Thrombocytopenia is associated with severe cases of dengue and most cases of fatality due to this infectious fever. Dengue virus reduces platelet count of the host directly or indirectly by changing the environment of bone marrow, influencing different factors involving platelet production, destroying and replicating into platelets and reducing the circulating platelets as suggested by different investigations. Though dengue has been observed to turn into epidemics every year in many countries with numerous cases of fatalities, preventive or curative treatment has yet to be found. Nonetheless, papaya leaves have been seen effective to improve the platelet count in investigations on dengue patients and *in vivo* animal models besides having larvicidal potential. Furthermore, several reports have suggested that papaya leaves have the ability to inhibit destructive effects on platelets by the dengue virus and increase the expression of ALOX 12 gene responsible for elevating platelet count. Moreover, papaya leaves have been found to have a wide therapeutic range with very few toxic effects. However, very few investigations were done to explore the mechanism(s) behind the role of papaya leaves to improve the platelet count. If the exact mechanisms are known, papaya leaves extract could be optimized for better effectiveness as well as therapeutic preparations that could be formulated targeting the same pathway as papaya leaves. Also, the anti-thrombocytopenic potential of papaya leaves is not very widely known. Since there is no randomized controlled clinical trial with significant patients available, its use is still neglected and not approved by the authorities including US-FDA. Thus, more clinical studies with a large number of patients should be conducted for the clinical and therapeutic role of papaya leaves as a curative agent in dengue to confirm and establish its use for dengue patients. Also, approved marketed preparation of papaya leaves in different dosage forms such as extract, syrup, tablet, etc. should be made available for dengue patients as a supplemental remedy, especially in countries with frequent dengue cases, to control the severity of this viral fever with multiple clinical complications and no remedy.

Currently available products based on Papaya leaves are lacking the standardization, dose optimization, therapeutic regimen, extensive/long term toxicity study reports, and proper approval of products from the regulatory authority. However, further research and monitoring on Papaya leaf-based products are required to overcome the above limitations.

We performed this comprehensive review to enlighten the scientific community about the therapeutic potential of Papaya leaves so that further analysis and investigations can be made to establish Papaya leaves preparations as a potential possible therapeutic agent for the effective and safe treatment of dengue fever; thus to prevent the death of many lives due to dengue fever, especially in developing countries. Therefore, extensive clinical trials and further investigations for the establishment of proper mechanism of action of Papaya leaves preparations along with the confirmation of its reported and identified bioactive compound(s) are required for the establishment of different preparations of *Carica papaya* L. leaves for the effective and safe treatment against dengue fever and to save thousands of lives around the world.
